# Stability in Crisis: Nurses’ Attitudes and Self-Efficacy Towards Caring for Patients With Multidrug-Resistant Bacteria During the Pandemic

**DOI:** 10.1177/00469580251332060

**Published:** 2025-06-10

**Authors:** Marte Johanne Tangeraas Hansen, Heidi Syre, Anne Marie Lunde Husebø, Marianne Storm, Ingvild Dalen

**Affiliations:** 1 Stavanger University Hospital, Norway

**Keywords:** attitudes, caring, infection prevention and control, infectious diseases, multidrug-resistant bacteria, nursing research, nursing workforce, pandemic, self-efficacy

## Abstract

Multidrug-resistant bacteria (MDRB) are microorganisms with global impact that also share modes of transmission with severe acute respiratory syndrome coronavirus 2. Nurses’ attitudes and self-efficacy towards caring for patients with MDRB are crucial in understanding their preventive behaviour, and a pandemic may acquire extraordinary Infection Prevention and Control (IPC) measures. To explore trends in nurses’ attitudes and self-efficacy when caring for patients with MDRB. This quantitative, prospective, longitudinal study used a repeated cross-sectional design. Nurses from 5 surgical wards and 2 oncology/haematology wards were invited to participate. The data were collected via 2 instruments: the Multidrug-Resistant Bacteria Attitude Questionnaire and the General Perceived Self-Efficacy Scale. The results were summarised with descriptive statistics, and longitudinal analyses were performed with mixed linear regression. No sample size calculations were made for this study. A total of 512 responses were received, the response rates for the time points were 60% (n = 131, T1), 32% (n = 72, T2), 47% (n = 109, T3), 48% (n = 108, T4), and 41% (n = 92, T5). No significant longitudinal changes in nurses’ attitudes and self-efficacy regarding infection prevention and control when caring for patients with MDRB were found. However, a small but significant negative change in nurses’ professional and emotional approach to caring for such patients was observed towards the end of the study period. A small but significant change in the nurses’ self-efficacy was observed between May 2020 and March 2021, indicating an increase in infection control self-efficacy during the first year of the COVID-19 pandemic. The stable knowledge, behavioural intentions and emotional responses contradict similar international studies. Nonetheless, moderate but stable emotional responses and high self-efficacy may indicate mental resilience in the nursing workforce, a pandemic preparedness resource that should be preserved.

## Introduction

The emerging challenge of multidrug-resistant bacteria (MDRB) is one of the most serious threats to public health.^1^ MDRB, such as methicillin-resistant *Staphylococcus aureus* (MRSA) and bacteria producing extended-spectrum beta-lactamase (ESBL), including carbapenem-resistant bacteria and vancomycin-resistant *Enterococcus* (VRE), are of global concern. Infections due to MDRB are challenging to cure, increasing the risk of increased disease burden and death.^2^ The impact and challenges that MDRB cause are known by many as the silent pandemic.^2,3^

The Corona Virus Disease 2019 (COVID-19) pandemic, caused by Severe Acute Respiratory Syndrome Corona Virus-2 (SARS-CoV-2), cannot be characterised as silent. It has been a prolonged worldwide crisis with a substantial global disease burden.^4,5^ During the COVID-19 pandemic, nurses endured a prolonged Infection Prevention and Control (IPC)-related emergency characterised by a lack of knowledge, equipment and preparedness^6,7^

SARS-CoV-2 and MDRB are both microorganisms with a global impact.^8^ They share modes of transmission through respiratory droplets and close contact.^9-11^ Patients in both disease populations are often isolated in a hospital setting to prevent transmission pathways in the hospital.^9^ IPC measures are implemented for both populations, including standard precautions, isolation precautions and personal protective equipment (PPE).^12,13^

Another aspect shared by the COVID-19 and silent MDRB pandemics is their impact on Health Care Workers’ (HCWs’) sense of safety. Numerous studies have found high levels of psychological distress among HCWs when caring for patients with SARS-CoV-2 infections, such as stress, anxiety, burnout and compassion fatigue related to IPC practices.^14-16^ These observations are consistent with studies examining nurses’ attitudes towards caring for patients with MDRB (both carriers and patients with ongoing infections), showing that HCWs, including nurses, face MDRB with insufficient knowledge and resources, resulting in stress, anxiety and anger.^17,18^

## Literature Review

A systematic review examining knowledge and attitude towards IPC standards found that despite adequate knowledge and positive attitudes, nurses had a low level of compliance with guidelines.^19^ Studies have also shown low adherence to IPC measures among HCWs during the COVID-19 pandemic.^20,21^ These studies indicate that low compliance with IPC measures among HCWs has been a challenge both before and during the COVID-19 pandemic.^11^

Nurses’ attitudes and self-efficacy towards caring for patients with MDRB are crucial in understanding their IPC behaviour. Attitudes, defined by Breckler^22^ as a response to an object, for example, the COVID-19 pandemic, contain 3 attitude components: cognition, behaviour and affect. Cognition refers to knowledge, behaviour includes observable actions, internal behavioural intentions or previous behaviour, while affect refers to the emotional response towards the attitude object.^22^ Self-efficacy refers to an individuals’ belief in their potential to produce a given outcome.^23^ Both positive attitudes and high self-efficacy are associated with robustness and resilience in nursing.^24^ Nonetheless, little is known about how the COVID-19 pandemic has influenced nurses’ attitudes and self-efficacy towards caring for isolated patients. Therefore, studying nurses’ attitudes and self-efficacy towards IPC is crucial for understanding their compliance with the given guidelines.

To our knowledge, no longitudinal studies have examined nurses’ attitudes and self-efficacy towards caring for patients with MDRB. Therefore, this study aimed to explore trends in nurses’ attitudes and self-efficacy when caring for patients with MDRB.

## Methods

This quantitative, prospective, longitudinal study used a repeated cross-sectional design, measuring nurses’ attitudes and self-efficacy when caring for isolated patients with MDRB.

### Context and Study Settings

This study was conducted at a Norwegian university hospital. The hospital is the workplace for approximately 2500 nurses.

#### The COVID-19 Pandemic

**Figure 1.**
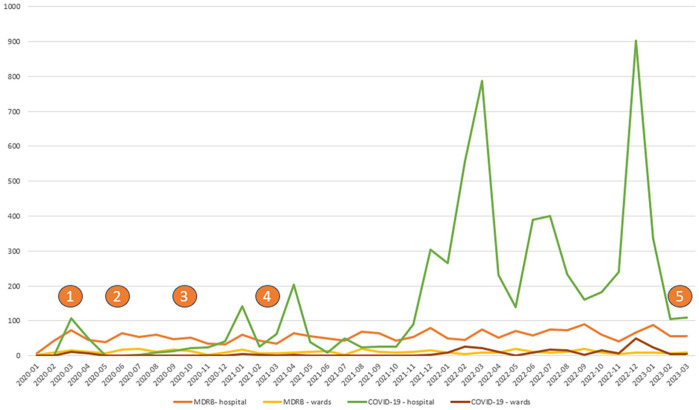
Longitudinal data was collected at 5 time points (T1-T5) during the COVID-19 pandemic.

As extraordinary IPC-measures played a major role in nurses’ clinical work during the pandemic, and thus during the study period, information on the COVID-19 pandemic provides an important backdrop for this study. This study was conducted between February 2020 and March 2023. The Norwegian Institute for Public Health started to test individuals for COVID-19 in January 2020. The first positive COVID-19 case in Norway was reported on 26 February 2020. The first suspected COVID-19 case in the participating hospital was admitted on 9 March 2020. From February 2020 to March 2021, 500 patients were admitted because of suspected or confirmed COVID-19 and most (n = 492) were admitted to the hospital’s medical wards. By May 2023, the number of patients admitted because of suspected or confirmed COVID-19 had increased to approximately 6400, of which 260 were admitted to the participating wards. Figure 1 shows the number of patients with suspected or confirmed COVID-19 at the hospital (green) and the participating wards (red) during the study period. These numbers were based on physicians’ diagnoses. IPC measures were initiated for all patients with suspected or confirmed COVID-19.

#### Antimicrobial Resistance

Norway has a low prevalence of antimicrobial resistance. The number of resistant isolates in Norway is persistently below the European average and is among the lowest globally.^25^ Approximately 430 patients with MRSA, ESBL-producing bacteria or VRE were admitted to the participating wards during the study period. The patients stayed for approximately 4000 days in total. Figure 1 shows the number of registered patients isolated due to MRSA, ESBL-producing bacteria and VRE infections at the hospital (orange) and the participating wards (yellow).

### Participants

Aiming to explore trends in nurses’ attitudes and self-efficacy when caring for patients with MDRD, our general sample was registered nurses. The inclusion criterions were that the nurses had to work in clinical, patient-related practice at 5 surgical wards and 2 Oncology/Haematology Wards (OHW), which all admit patients with MDRB. These wards were picked out by convenience, as the other eligible wards at the hospital was part of a different project at the time of the first data collection. The exclusion criteria were nurses in clinical practice without a bachelor’s degree in nursing (including nursing students), in administrative positions, conducting outpatient work or absent during the data collection period due to sick leave, holidays or parental leave. All eligible nurses were invited to participate by e-mail. No sample size calculations were made for this study.

### Data Collection

Data was collected in February-March 2020 (T1), May 2020 (T2), October 2020 (T3), February-March 2021 (T4), and February-March 2023 (T5; Figure 1) using Corporator Surveyor, a software program accessed through the hospital’s Information, Communication and Technology Department. The data collection at T5 was decided after the 4 first data collections was performed, to gain a more comprehensive data material.

### Instruments

The survey, consisting of 2 questionnaires, was checked for face validity by 8 experienced nurses, 6 working as practice development nurses in the participating wards and 2 working as IPC nurses. The survey was revised based on their responses. It took about 20 minutes to complete the questionnaires. The demographic data included sex, age, education level, work orientation and work experience. T1 was initiated before the outbreak of the COVID-19 pandemic in Norway. In T2, the demographic questionnaire was adjusted to include a question on experience working on a COVID-19 ward.

#### Attitudes

The Multidrug-Resistant Bacteria Attitude Questionnaire (MDRBAQ)^26,27^ is based on the 3-component model of attitudes (knowledge, behaviour and emotional response) and has been used in previous studies.^18,28,29^ The psychometric properties of the MDRBAQ have been found satisfying.^26^

The knowledge component comprises 25 yes/no and multiple-choice questions designed to reveal nurses’ knowledge on topics related to caring for patients with MDRB. The questionnaire was updated according to current Norwegian IPC and antibiotic stewardship guidelines, and 4 questions on antibiotics were added. Questions answered as ‘I don’t know’ or unanswered were considered incorrect. Scores range from 0 (no correct answers) to 25 (all correct answers).^18^

The behavioural component covered the topics of standard precautions, microbiological testing, isolation precautions and antibiotic stewardship, and it was updated according to current Norwegian IPC and antibiotic stewardship guidelines. The participants were asked to indicate their agreement after reading a clinical case made in collaboration with the hospital’s IPC Department. This component was answered using a six-point Likert scale ranging from totally agree to totally disagree, with an ‘I don’t know’ option. Totally agreeing or agreeing was viewed as correct behaviour and given 1 point. Disagreeing or not knowing was viewed as incorrect and given no points. Scores range from 0 to 24.

The emotional response component, which is a scale validated in earlier research,^26^ remained unchanged. It contains 14 items answered using a seven-point Likert scale, with the scores ranging from 14 to 98. Lower scores indicated more negative emotions. The emotional response scale is divided into 3 subscales^27^: competence (6 items, sum score of 6-42), professional approach (5 items, sum score of 5-35) and mood (3 items, sum score of 3-21; Table 2). The Cronbach’s α of the MDRBAQ emotional response component was between .86 and .90 for the 5 time points (Supplemental Table S1).

#### Self-Efficacy

Nurses’ self-efficacy was assessed using the Norwegian version of the 10-item General Perceived Self-Efficacy Scale.^30^ Previous results using the Norwegian version of the General Perceived Self-efficacy scale have shown that the factor structure, internal consistency and test-retest reliability of the scale were satisfactory.^30^ This scale comprises 10 statements answered on a 4-point Likert scale, with sum scores ranging from 10 to 40. The participants responded after reading a clinical case concerning a patient with MDRB. The Cronbach’s α was between .83 and .88 for the 5 time points (Supplemental Table S1).

### Data Analysis

The data were analysed using SPSS software (version 13; SPSS Inc., Chicago, IL, USA). The demographic data, clinical data and subscale scores were summarised using descriptive statistics, including counts (percentages) for categorical variables, medians (interquartile ranges [IQR]) for skewed continuous variables, and means (standard deviations [SD]) for symmetrically distributed continuous variables. Six outliers with non-logical responses were excluded from the self-efficacy analysis.

Longitudinal analyses were performed in Stata software (version 17; StataCorp, College Station, TX, USA) using mixed linear regression with time point as a categorical fixed effect and a random intercept and slope for time to allow for correlations between the repeated measurements of the same respondents. The outcomes were entered on a transformed, common scale (0-100). The results are presented as predicted means with 95% confidence intervals (CIs). Selected comparisons between time points were performed with Wald tests. Adjusted analyses included age group, sex, years since completed education, specialist nurse (yes/no), working on an OHW (yes/no) and having ever worked on a pandemic ward (yes/no) as covariates. Adjusted predicted means were estimated for the following groups: aged 26 to 30 years, female sex, 5 years since completed education, not a specialist, not working on an OHW and no experience working on a pandemic ward.

The main analyses included all available cases. Supplementary analyses were performed that included only respondents for whom we had baseline data. A *P*-value of <.05 was considered statistically significant.

We used the STROBE cross sectional checklist when writing our report.^31^

### Ethical Considerations

This study was performed according to the Declaration of Helsinki and appraised and approved by the hospitals’ privacy commissioner (approval IDs 807, January 2020 and 1515, May 2020). Written informed consent to participate in the study was obtained from all participants.

## Results

### Demographics

Five hundred twelve responses were received across the 5 time points from 256 unique nurses. One hundred thirty-six (36%) nurses participated more than once, with 10 participating at all 5 time points. The population was predominantly female, and over 60% of the participants were in the 18 to 35 age group at all time points (Table 1). The response rates for the time points were 60% (n = 131, T1), 32% (n = 72, T2), 47% (n = 109, T3), 48% (n = 108, T4) and 41% (n = 92, T5).

**Table 1.** Study Sample Characteristics.

Characteristic	T1 (n = 131)	T2 (n = 72)	T3 (n = 109)	T4 (n = 108)	T5 (n = 92)
Age (years)					
<26	25 (19%)	15 (21%)	27 (25%)	26 (24%)	20 (22%)
26-30	36 (28%)	17 (24%)	36 (33%)	32 (30%)	30 (33%)
31-35	22 (17%)	15 (21%)	18 (17%)	22 (20%)	15 (16%)
36-40	12 (9%)	8 (11%)	6 (6%)	5 (5%)	8 (9%)
41-45	6 (5%)	5 (7%)	4 (4%)	5 (5%)	4 (4%)
>45	29 (22%)	12 (17%)	18 (17%)	18 (17%)	15 (16%)
Missing	1	-	-	-	-
Sex					
Female	125 (95%)	68 (94%)	99 (91%)	101 (94%)	86 (93%)
Male	6 (5%)	4 (6%)	10 (9%)	7 (6%)	6 (7%)
Years since completed education					
Median (IQR)	8 (4, 13)	7.5 (3.5, 13)	5 (2, 10)	6 (2, 11)	5 (2, 12)
0-5	52 (39%)	26 (36%)	59 (54%)	53 (49%)	47 (51%)
6-10	36 (27%)	24 (33%)	26 (24%)	23 (21%)	17 (18%)
11-15	14 (11%)	7 (10%)	6 (6%)	11 (10%)	12 (13%)
16-20	8 (6%)	5 (7%)	5 (5%)	6 (6%)	4 (4%)
>20	22 (17%)	10 (14%)	13 (12%)	15 (14%)	12 (13%)
Specialist					
Yes	34 (26%)	25 (35%)	25 (23%)	24 (22%)	18 (20%)
No	97 (74%)	47 (65%)	84 (77%)	84 (78%)	74 (80%)
OHW					
Yes	45 (34%)	34 (47%)	37 (34%)	32 (30%)	31 (34%)
No	86 (66%)	38 (53%)	72 (66%)	76 (70%)	61 (66%)
Experience on a pandemic ward					
Yes	0 (0%)	3 (4%)	16 (15%)	12 (11%)	11 (12%)
No	131 (100%)	69 (96%)	93 (85%)	96 (89%)	79 (88%)
Missing	-	-	-	-	2

*Note*. All results are presented as the count (percentage) unless otherwise specified.

IQR = interquartile range; OHW = oncology and haematology ward.

### Attitudes and Self-Efficacy

Table 2 presents the means and SDs of all measures at each time point. Mean scores varied from 18.8 (T3) to 19.3 (T2) for knowledge, 18.2 (T2) and 18.6 (T3) for behaviour, 72.0 (T5) and 75.8 (T2) for emotional response, and 31.2 (T2) and 31.8 (T4) for self-efficacy (Table 2). Overall, the scores observed over the study period were stable for all measures. In the self-efficacy scale 6 outliers were removed due to them seeming illogical in the context of the participants’ other answers.

**Table 2.** The Mean (SD) of Scores Reflecting Nurses’ Attitudes and Self-Efficacy Towards Caring for Patients With MDRB at Each Time Point.

Variable	Range	T1 (n = 131)	T2 (n = 72)	T3 (n = 109)	T4 (n = 108)	T5 (n = 92)
Knowledge	0-25	18.9 (2.2)^n = 116^	19.3 (2.3)^n = 63^	18.8 (2.5)^n = 98^	18.9 (2.6)^n = 101^	18.9 (2.3)^n = 85^
Behaviour	0-24	18.2 (2.4)^n = 126^	18.4 (2.3)^n = 69^	18.6 (2.6)^n = 107^	18.3 (2.6)^n = 104^	18.4 (2.4)^n = 87^
Emotional response	14-98	74.4 (11.0)^n = 116^	75.8 (10.5)^n = 69^	75.3 (10.3)^n = 103^	74.7 (11.0)^n = 105^	72.0 (12.1)^n = 81^
Professional	6-42	33.1 (5.1)^n = 121^	33.3 (4.8)^n = 71^	33.3 (4.8)^n^ ^= 104^	32.5 (5.3)^n = 107^	31.3 (5.5)^n = 84^
Competence	5-35	26.6 (5.3)^n = 126^	27.3 (5.0)^n = 71^	26.9 (4.9)^n = 106^	27.5 (5.0)^n = 106^	26.5 (5.4)^n = 89^
Mood	3-21	14.6 (3.3)^n = 127^	15.1 (2.9)^n = 71^	15.0 (3.1)^n = 107^	14.9 (2.9)	14.4 (3.4)
Self-efficacy*	10-40	31.6 (3.5)^n = 126^	31.2 (3.5)^n = 69^	31.8 (3.5)^n = 107^	31.8 (3.7)^n = 104^	31.4 (3.6)^n = 86^

* Six outliers were removed. SDs are given in parentheses, with the number of valid observations shown in superscript.

The mixed linear regression analysis (Table 3) showed no substantial changes in nurses’ knowledge and behaviour during the study period. A significant positive change was observed in the nurses’ self-efficacy between T2 and T4 (*P* = .041), indicating an increase in self-efficacy during the first year of the COVID-19 pandemic. In addition, the mean scores for the ‘professional approach’ emotional response subscale differed significantly between T1 and T5 (*P* = .028) and between T2 and T5 (*P* = .012), indicating that the nurses experienced a decrease in their emotional, professional approach towards patients with MDRB during the study period. Similar results were obtained after adjusting for covariates (Figure 3 and Table 3, Supplemental Figures S1 and S2). Supplementary analyses that included only respondents who also participated in the T1 survey are presented in Supplemental Table S2 and Supplemental Figures S3 to S6.

**Table 3.** Observed and Predicted (Adjusted) Mean Scores of Responses Given in Percent of Max Score at Each Time Point, With Selected Comparisons.

Model/Outcome		T1	T2	T3	T4	T5	T2 vs T1	T5 vs T1	T4 vs T2	T5 vs T2
Unadjusted	n/obs	Mean (95% CI)	Mean (95% CI)	Mean (95% CI)	Mean (95% CI)	Mean (95% CI)	*P*	P	P	P
Knowledge	239/463	75.0 (73.4, 76.6)	76.1 (74.1, 78.1)	75.0 (73.3, 76.7)	75.1 (73.4, 76.8)	75.4 (73.5, 77.4)	.34	.70	.41	.62
Behaviour	250/493	76.0 (74.4, 77.6)	76.5 (74.5, 78.5)	76.8 (75.1, 78.5)	75.4 (73.6, 77.2)	76.0 (74.0, 78.1)	.62	.96	.35	.71
Emotional response	241/474	75.0 (73.3, 76.7)	76.3 (74.3, 78.3)	75.4 (73.6, 77.2)	75.2 (73.3, 77.1)	74.2 (72.0, 76.4)	.22	.52	.32	.11
Professional	247/487	78.0 (76.1, 79.9)	78.7 (76.4, 81.0)	78.2 (76.1, 80.2)	76.8 (74.7, 78.9)	74.9 (72.5, 77.4)	.56	**.028**	.14	**.012**
Competence	251/498	75.1 (72.8, 77.3)	76.2 (73.5, 79.0)	74.8 (72.4, 77.2)	76.6 (74.2, 79.0)	75.8 (73.2, 78.3)	.41	.64	.80	.77
Mood	254/505	68.9 (66.6, 71.3)	71.3 (68.4, 74.2)	70.8 (68.3, 73.3)	69.7 (67.1, 72.3)	69.9 (67.1, 72.8)	.15	.56	.35	.48
Self-efficacy*	249/492	78.8 (77.4, 80.1)	77.5 (75.7, 79.2)	79.4 (77.9, 80.9)	79.6 (78.0, 81.1)	78.5 (76.7, 80.2)	.18	.78	**.041**	.37
Adjusted	n/obs	Marginal mean (95% CI)	Marginal mean (95% CI)	Marginal mean (95% CI)	Marginal mean (95% CI)	Marginal mean (95% CI)	P	P	P	P
Knowledge	236/460	75.2 (73.0, 77.5)	76.4 (73.7, 79.0)	75.5 (73.1, 77.9)	75.6 (73.3, 77.9)	75.9 (73.4, 78.4)	.31	.55	.51	.72
Behaviour	247/490	73.4 (71.1, 75.7)	74.1 (71.4, 76.8)	74.8 (72.3, 77.2)	73.3 (70.9, 75.7)	73.7 (71.0, 76.3)	.51	.83	.49	.72
Emotional response	239/472	73.9 (71.5, 76.4)	75.1 (72.3, 77.9)	74.0 (71.5, 76.6)	73.9 (71.3, 76.5)	72.7 (69.9, 75.6)	.27	.33	.28	.069
Professional	244/484	76.8 (74.1, 79.5)	77.6 (74.5, 80.6)	77.0 (74.2, 79.8)	75.7 (72.9, 78.5)	73.7 (70.6, 76.8)	.56	**.029**	.16	**.011**
Competence	249/496	73.8 (70.6, 77.0)	74.9 (71.3, 78.6)	73.3 (70.0, 76.7)	75.1 (71.8, 78.4)	74.0 (70.6, 77.5)	.43	.89	.91	.58
Mood	251/502	68.7 (65.3, 72.0)	70.7 (66.8, 74.6)	70.2 (66.6, 73.7)	69.2 (65.7, 72.7)	69.7 (65.9, 73.4)	.21	.56	.39	.60
Self-efficacy*	247/490	77.1 (75.1, 79.1)	75.6 (73.2, 77.9)	77.4 (75.3, 79.5)	77.6 (75.5, 79.7)	76.5 (74.3, 78.8)	.11	.61	**.042**	.38

*Note*. The adjustment variables are fixed at age group = 26-30 years, gender = female, years since finished education = 5, specialist = no, OHW = no, experience from pandemic post = no. Bold numbers represent statistical significance.

* Six outliers excluded for self-efficacy.

## Discussion

Our prospective, longitudinal study examined trends in hospital nurses’ attitudes and self-efficacy when caring for patients with MDRB. There was no evidence of significant longitudinal changes in nurses’ attitudes and self-efficacy, including knowledge and behaviours regarding IPC when caring for patients with MDRB (Table 2). Our findings are somewhat unexpected given the similarity in IPC measures for MDRB and SARS-CoV-2 and the increased focus on such measures during the COVID-19 pandemic. One possible explanation for this stability is high baseline attitudes and self-efficacy in the studied population, which can be associated with the relatively high educational level of Norwegian HCWs.^32^ Another possible explanation is that the participants in our study were nurses who usually cared for patients with MDRB and, thus, have been continuously exposed to information and practices related to MDRB.

Our study found that nurses had moderate emotional responses towards caring for patients with MDRB. No significant longitudinal changes were observed in their general emotional responses during the COVID-19 pandemic, indicating robustness and stability in nurses’ emotional responses. This finding was surprising since many international articles have reported that fear, anxiety and stress had major effects on nurses’ psychological well-being.^15^ During the first wave of the COVID-19 pandemic, there was ambiguous information about the contagion and a lack of PPE, and guidelines changed nearly daily.^14^ Nurses also reported insufficient training, lack of self-care, physical discomfort wearing PPE, fear and anxiety,^14,15,33^ as was also the case in Norway. However, Norway’s approach towards the COVID-19 pandemic was largely a success, and Norway was among the European countries with the lowest mortality rate, the lowest burden of measures and the smallest reduction in economic activity due to the COVID-19 pandemic.^34,35^ These observations suggest that the nurses working in a clinical setting in Norway during the COVID-19 pandemic experienced less COVID-19-related stress and uncertainty and a greater sense of safety than nurses in other countries. Furthermore, caring for patients with MDRB may be perceived as more familiar and safer than caring for patients with SARS-CoV-2 infections.

Regarding the participating nurses’ emotional responses when caring for patients with MDRB, our data (Table 3) showed a small but significant negative change in the ‘professional approach’ subcategory between T1 (before the COVID-19 pandemic) and T5 (late pandemic phase) and between T2 (during the first wave) and T5 (late pandemic phase). Therefore, our findings suggest that the nurses experienced a decrease in their emotional, professional approach during the COVID-19 pandemic. This subcategory contains emotions such as interest, engagement and concentration. Hospital records from the same period suggest a massive increase in patients with suspected or confirmed COVID-19 in the participating wards, especially from T4 to T5. There were no observable changes in the number of patients with MDRB (Figure 1). The high number of patients isolated in the wards around T5 may explain the significant change. Caring for isolated patients can take an emotional toll. One study found that nurses experience ambiguous emotions towards caring for patients with MDRB.^18^ This finding is also supported by Nila and Roxsana Devi,^36^ which found a significant relationship between nurses’ stress and fatigue levels while treating isolated patients with COVID-19.^36^ Supportive leadership, debriefing and setting professional boundaries are suggested as preventive interventions.^37,38^

**Figure 2.**
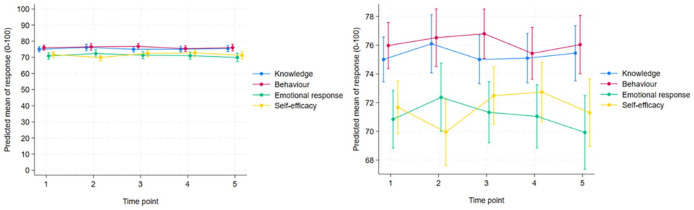
Means scores (0-100 scale) for knowledge, behaviour, emotional response and self-efficacy at time points T1-T5 with 95% CIs (whiskers). The right-hand plot is a zoomed-in version of the left-hand plot.

In contrast to the slight decline in the nurses’ emotional, professional approach, their self-efficacy was high throughout the study period, indicating that they had a firm belief in their capability to organise and execute the courses of action needed to successfully care for patients with MDRB.^23^ The small but significant increase in self-efficacy during the first year of the COVID-19 pandemic (Table 3, Figure 2) may indicate that the nurses’ belief in their abilities was higher after the first year than during the first wave. High self-efficacy can be interpreted as an important resource for pandemic preparedness. Self-efficacy may have a mediating effect on work-related stress and lead to work resilience.^24,39^ Positive psychological resources associated with retaining self-efficacy and resilience can help preserve nurses’ mental health^40^ and thus reduce the negative impact associated with healthcare crises. According to Bandura,^23^ sources of nurses’ self-efficacy are success, good role models, encouragement and caring for their mental status. Consequently, they are important when making contingency plans for future healthcare crises.

**Figure 3.**
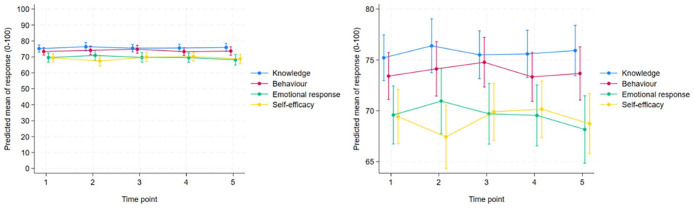
Predicted mean scores (0-100 scale) for knowledge, behaviour, emotional response and self-efficacy at time points T1 to T5. The adjustment variables were fixed at age grou*P* = 26-30 years, sex = female, years since completed education = 5, specialist = no, OHW = no, and experience on a pandemic ward = no. The right-hand plot is a zoomed-in version of the left-hand plot. Six outliers were excluded for self-efficacy.

As previously stated, we found no significant change in self-reported knowledge and behaviour when caring for patients with MDRB. One study in China showed that HCWs generally reported better IPC behaviours during than before the COVID-19 pandemic.^11^ Huang et al^41^ found increased compliance with hand hygiene during the first wave of the COVID-19 pandemic, but compliance reduced over time. A Danish study also found this trend.^21^ Huang et al.^41^ explained the increase in self and patient protection due to fear of crisis in the first wave, and Stangerup et al.^21^ suggested that nurses return to old routines once they perceive the risk as reduced. Therefore, our findings may suggest that the participating nurses perceived the risk of infection as relatively stable both before and during the COVID-19 pandemic. Jeong and Eun^42^ found that nurses’ performance confidence affected their infection control practices. Therefore, high self-efficacy and self-reported behaviour suggest high compliance among the studied nurses.

## Strengths and Limitations

Our study had several notable strengths. Firstly, it involved a substantial number of participants, with 512 responses from 256 unique nurses. Secondly, it used validated and structured questionnaires.

However, it also had some limitations that should be noted. Firstly, self-reporting of compliance rates tends to overestimate the actual rates. However, we are convinced that the information given to the participants and the anonymity of the questionnaires gave them the freedom to describe their attitudes and self-efficacy and increased our study’s credibility. Secondly, no sample size calculations were made for this study, which may have affected the accuracy of the results. A proper sample size calculation can ensure enough power to detect meaningful changes over time, and that the study sample is representative of the broader population.^43^ Thirdly, our study’s internal validity may have been impacted by selection bias, given the 32% to 60% response rate.^44^ For instance, we don’t know whether the motivation to respond to the survey differed between the various time points, or if respondents were motivated to participate for different reasons at each time point. The results were adjusted for age group, sex, years since completed education, specialist nurse (yes/no), working on an OHW (yes/no) and having ever worked on a pandemic ward (yes/no), which may have increased the generalisability of the results. Lastly, the studys’ validity may be impacted by the retest effect^44^ with 136 (36%) nurses participating more than once and 10 participating in all 5 surveys. However, our results showed stability, indicating that any retest effect was marginal.

## Implications for Practice

The participating nurses showed stability in IPC knowledge and behavioural intentions, stable emotional responses and high self-efficacy. These positive resources can reduce the impact of IPC crises, and measures to retain them should be integral to contingency plans for future healthcare crises. A small negative change in nurses’ professional emotions when caring for isolated patients was observed when the number of isolation beds increased. Interventions to prevent negative emotions include leadership, debriefing and setting professional boundaries.

## Conclusions

This prospective, longitudinal study examined trends in hospital nurses’ attitudes and self-efficacy when caring for patients with MDRB. There was no evidence of significant longitudinal changes in nurses’ attitudes and self-efficacy regarding IPC. Our data showed a small but significant negative change in nurses’ professional approach, suggesting that they experienced more negative professional emotions towards caring for patients with MDRB during the late COVID-19 pandemic phase. It also showed a small but significant change in nurses’ self-efficacy from May 2020 to March 2021, indicating an increase in self-efficacy during the first year of the COVID-19 pandemic. Our findings are meaningful when making contingency plans for future healthcare crises.

## Supplemental Material

sj-docx-1-inq-10.1177_00469580251332060 – Supplemental material for Stability in Crisis: Nurses’ Attitudes and Self-Efficacy Towards Caring for Patients With Multidrug-Resistant Bacteria During the Pandemic Supplemental material, sj-docx-1-inq-10.1177_00469580251332060 for Stability in Crisis: Nurses’ Attitudes and Self-Efficacy Towards Caring for Patients With Multidrug-Resistant Bacteria During the Pandemic by Marte Johanne Tangeraas Hansen, Heidi Syre, Anne Marie Lunde Husebø, Marianne Storm and Ingvild Dalen in INQUIRY: The Journal of Health Care Organization, Provision, and Financing

sj-docx-2-inq-10.1177_00469580251332060 – Supplemental material for Stability in Crisis: Nurses’ Attitudes and Self-Efficacy Towards Caring for Patients With Multidrug-Resistant Bacteria During the Pandemic Supplemental material, sj-docx-2-inq-10.1177_00469580251332060 for Stability in Crisis: Nurses’ Attitudes and Self-Efficacy Towards Caring for Patients With Multidrug-Resistant Bacteria During the Pandemic by Marte Johanne Tangeraas Hansen, Heidi Syre, Anne Marie Lunde Husebø, Marianne Storm and Ingvild Dalen in INQUIRY: The Journal of Health Care Organization, Provision, and Financing
