# Supplementary material for: Stability in Crisis: Nurses’ Attitudes and Self-Efficacy Towards Caring for Patients With Multidrug-Resistant Bacteria During the Pandemic
Source: Inquiry. 2025 Jun 10;62:00469580251332060. doi: 10.1177/00469580251332060 (PMC12159472; doi:10.1177/00469580251332060)
Supplement: sj-docx-2-inq-10.1177_00469580251332060 – Supplemental material for Stability in Crisis: Nurses’ Attitudes and Self-Efficacy Towards Caring for Patients With Multidrug-Resistant Bacteria During the Pandemic [file sj-docx-2-inq-10.1177_00469580251332060.docx]

**SUPPLEMENTARY TABLES AND FIGURES**

**Supplementary Table S1.** Cronbach’s α for the emotional response and self-efficacy scales at the five time points.

|  | T1  (*n* = 131) | T2  (*n* = 72) | T3  (*n* = 109) | T4  (*n* = 108) | T5  (*n* = 92) |
| --- | --- | --- | --- | --- | --- |
| Emotional response (14 items) | 0.88 | 0.88 | 0.86 | 0.90 | 0.89 |
| Self-efficacy (10 items) | 0.87 | 0.93 | 0.84 | 0.94 | 0.85 |
| Self-efficacy* | 0.83 | 0.85 | 0.84 | 0.88 | 0.85 |

* Excluding the six outliers.

**Supplementary table S2.** Subset from the beginning

| Model/Outcome |  | T1 | T2 | T3 | T4 | T5 | T2 vs T1 | T5 vs T1 | T4 vs T2 | T5 vs T2 |
| --- | --- | --- | --- | --- | --- | --- | --- | --- | --- | --- |
| *Unadjusted* | *n*/obs | mean  (95% CI) | mean  (95% CI) | mean  (95% CI) | mean  (95% CI) | mean  (95% CI) | *p* | *p* | *p* | *p* |
| Knowledge | 124/307 | 75.2 (73.6, 76.9) | 75.9 (73.7, 78.2) | 75.6 (73.4, 77.7) | 75.1 (72.8, 77.4) | 78.3 (75.6, 81.1) | 0.56 | **0.028** | 0.57 | 0.13 |
| Behaviour | 130/325 | 76.0 (74.3, 77.7) | 76.7 (74.4, 79.0) | 76.4 (74.1, 78.6) | 75.4 (73.0, 77.8) | 77.6 (74.7, 80.5) | 0.56 | 0.28 | 0.34 | 0.57 |
| Emotional response | 125/309 | 71.8 (69.5, 74.1) | 73.8 (70.9, 76.7) | 73.1 (70.2, 76.0) | 72.3 (69.1, 75.4) | 72.3 (68.5, 76.2) | 0.13 | 0.76 | 0.33 | 0.45 |
| Professional | 128/318 | 75.0 (72.6, 77.5) | 76.5 (73.2, 79.7) | 75.6 (72.4, 78.9) | 74.4 (70.9, 78.0) | 71.9 (67.4, 76.3) | 0.34 | 0.14 | 0.27 | **0.043** |
| Competence | 129/326 | 72.4 (69.4, 75.3) | 73.6 (69.9, 77.4) | 73.1 (69.5, 76.8) | 74.3 (70.5, 78.1) | 75.1 (70.8, 79.4) | 0.45 | 0.17 | 0.72 | 0.52 |
| Mood | 129/329 | 64.4 (61.3, 67.4) | 68.2 (64.1, 72.3) | 67.3 (63.2, 71.4) | 64.1 (59.7, 68.5) | 68.2 (62.8, 73.6) | 0.052 | 0.15 | 0.084 | 0.99 |
| Self-efficacy* | 129/321 | 72.0 (70.1, 73.9) | 70.0 (67.3, 72.7) | 73.4 (70.8, 76.0) | 73.5 (70.7, 76.4) | 72.4 (68.9, 75.9) | 0.15 | 0.81 | **0.035** | 0.22 |
|  |  |  |  |  |  |  |  |  |  |  |
| *Adjusted* | *n*/obs | marginal mean (95% CI) | marginal mean (95% CI) | marginal mean (95% CI) | marginal mean (95% CI) | marginal mean (95% CI) | *p* | *p* | *p* | *p* |
| Knowledge | 123/306 | 75.4 (72.8, 78.0) | 76.0 (73.0, 79.1) | 75.7 (72.7, 78.7) | 75.1 (72.0, 78.1) | 78.4 (74.7, 82.0) | 0.59 | **0.043** | 0.48 | 0.15 |
| Behaviour | 129/324 | 73.5 (70.9, 76.2) | 74.5 (71.4, 77.6) | 74.4 (71.4, 77.4) | 73.1 (69.9, 76.2) | 74.1 (70.3, 77.8) | 0.39 | 0.70 | 0.29 | 0.80 |
| Emotional response | 124/308 | 68.9 (65.3, 72.4) | 70.2 (66.2, 74.2) | 69.1 (65.1, 73.1) | 68.2 (64.0, 72.4) | 68.1 (63.0, 73.3) | 0.31 | 0.69 | 0.19 | 0.29 |
| Professional | 127/317 | 72.5 (68.7, 76.3) | 73.7 (69.2, 78.1) | 72.5 (68.1, 77.0) | 71.3 (66.6, 76.0) | 68.5 (62.7, 74.3) | 0.44 | 0.083 | 0.20 | **0.029** |
| Competence | 128/325 | 69.1 (64.5, 73.7) | 69.7 (64.4, 74.9) | 68.8 (63.8, 73.9) | 69.8 (64.6, 75.0) | 70.5 (64.4, 76.5) | 0.73 | 0.51 | 0.94 | 0.73 |
| Mood | 128/328 | 61.3 (56.5, 66.1) | 64.1 (58.4, 69.7) | 63.0 (57.4, 68.6) | 60.0 (54.2, 65.8) | 63.9 (56.9, 70.9) | 0.16 | 0.33 | 0.085 | 0.96 |
| Self-efficacy* | 128/320 | 68.6 (65.5, 71.7) | 66.2 (62.4, 69.9) | 69.5 (65.9, 73.1) | 69.8 (66.1, 73.5) | 68.5 (64.1, 72.9) | 0.077 | 0.95 | **0.028** | 0.23 |

The adjustment variables were fixed at age group = 26–30 years, sex = female, years since completed education = 5, specialist = no, OHW = no, experience working on a pandemic ward = no. *Six outliers were excluded for self-efficacy. Bold numbers represent statistical significance.

| 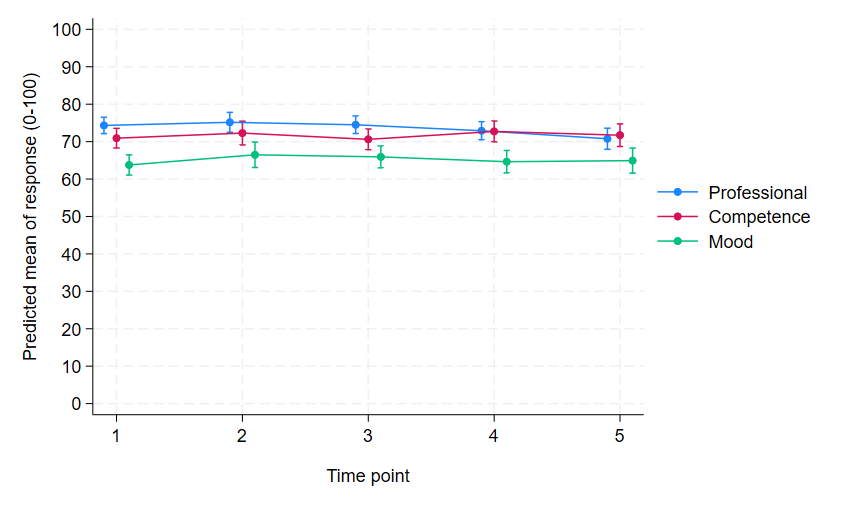 | 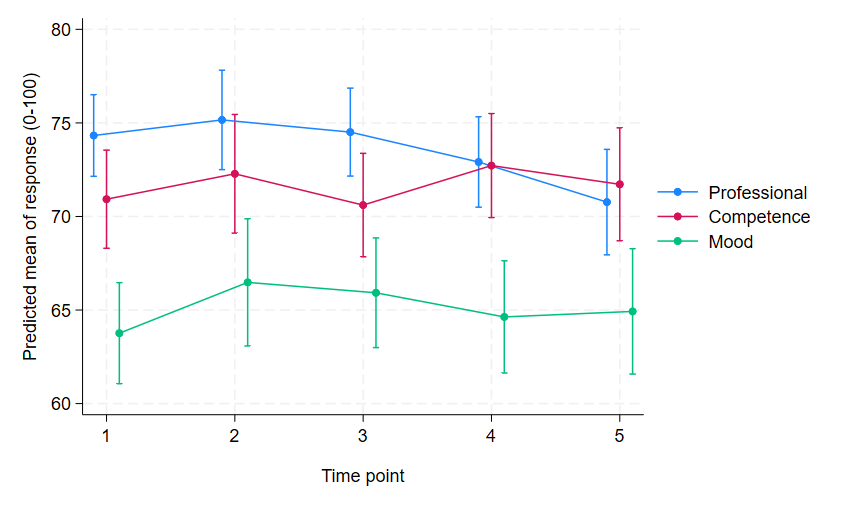 |
| --- | --- |

**Supplementary Figure S1.** The mean scores given as a percentage of the maximum for the emotional response subscales of professional, competence and mood at each time point with 95% CIs (whiskers). The right-hand plot is a zoomed-in version of the left-hand plot.

| 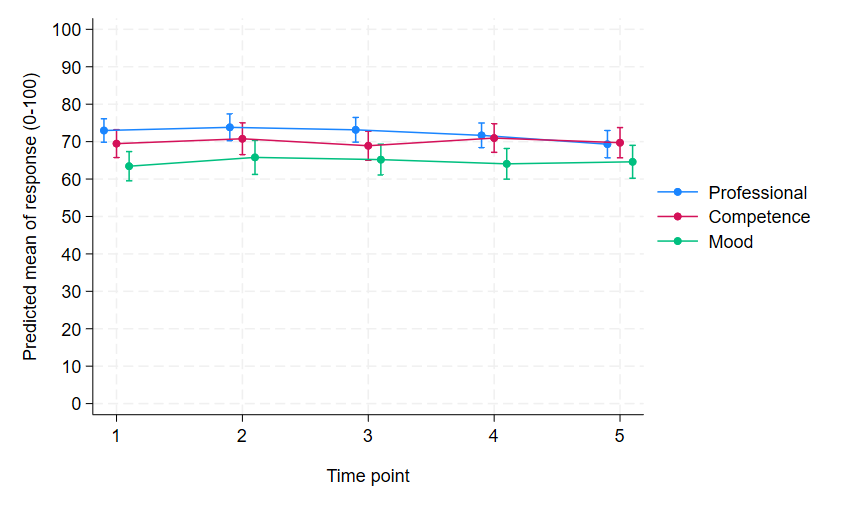 | 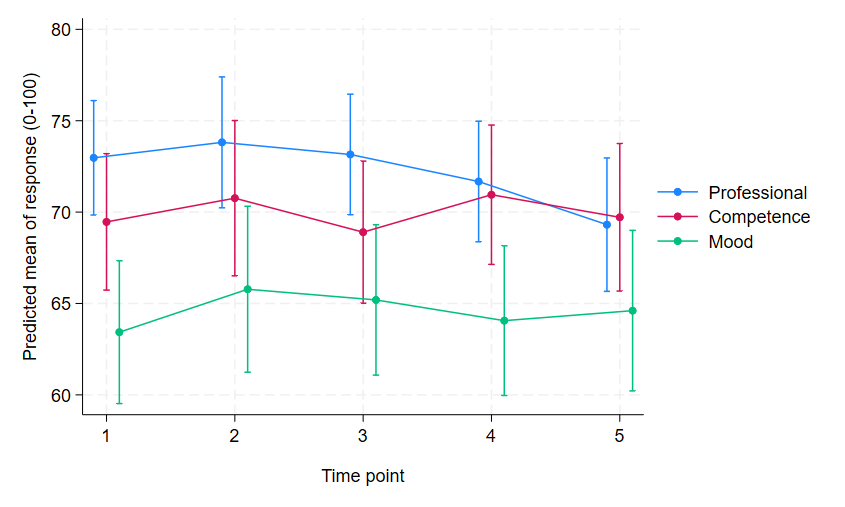 |
| --- | --- |

**Supplementary Figure S2.** Predicted mean scores given as a percentage of the maximum score for the emotional response subscales of professional, competence and mood at each time point with 95% Cis. The adjustment variables were fixed at age group = 26–30 years, sex = female, years since completed education = 5, specialist = no, OHW = no, experience on a pandemic ward = no. The right-hand plot is a zoomed-in version of the left-hand plot.

|  |  |
| --- | --- |

**Supplementary Figure S3.** Mean scores given as a percentage of the maximum for knowledge, behaviour, emotional response and self-efficacy at each time point for the subset of participants included at T1 with 95% CIs (whiskers). The right-hand plot is a zoomed-in version of the left-hand plot. No significant differences were observed over time in any of the measures. Six outliers were excluded for self-efficacy.

|  |  |
| --- | --- |

**Supplementary Figure S4.** Mean scores given as a percentage of the maximum score for the emotional response subscales of professional, competence and mood at each time point for the subset of participants included at T1 with 95% CIs (whiskers). The right-hand plot is a zoomed-in version of the left-hand plot.

|  |  |
| --- | --- |

**Supplementary Figure S5.** Predicted mean scores given as a percentage of the maximum for knowledge, behaviour, emotional response and self-efficacy at each time point for the subset of participants included at T1 with 95% CIs (whiskers). The adjustment variables were fixed at age group = 26–30 years, sex = female, years since completed education = 5, specialist = no, OHW = no, experience on a pandemic ward = no. The right-hand plot is a zoomed-in version of the left-hand plot. No significant differences were observed over time in any of the measures. Six outliers were excluded for self-efficacy.

|  |  |
| --- | --- |

**Supplementary Figure S6.** Predicted mean scores given as a percentage of the maximum for the emotional response subscales of professional, competence and mood at each time point for the subset of participants included at T1 with 95% CIs (whiskers). The adjustment variables were fixed at age group = 26–30 years, sex = female, years since completed education = 5, specialist = no, OHW = no, experience on a pandemic ward = no. The right-hand plot is a zoomed-in version of the left-hand plot.
